# Pandemic and epidemic intelligence innovation forum: bridging gaps in epidemic intelligence through global collaboration

**DOI:** 10.1186/s12919-025-00316-6

**Published:** 2025-02-28

**Authors:** Barbara Tornimbene, Zoila Beatriz Leiva Rioja, Oliver Morgan

**Affiliations:** 1 World Health Organization Hub for Pandemic and Epidemic Intelligence, Berlin, Germany; 2 CPC Analytics, Berlin, Germany

The practice of public health surveillance and epidemic intelligence is undergoing rapid evolution due to a remarkable and fast-paced innovations in laboratory science and data science [1]. These innovations are increasingly pushing the practice of epidemic intelligence to be ever more cross-disciplinary and globally networked [1, 2]. The pace of this evolution has only quickened as a result of the COVID-19 pandemic [3]. To maximize the opportunities arising from such rapid changes, public health scientists and practitioners need a coordinated and transparent information -sharing mechanism to learn about innovations and opportunities available across different professional sectors and from different parts of the world [1, 4, 5]. To provide this mechanism, the WHO Hub for Pandemic and Epidemic Intelligence established an Innovation Forum, a quarterly exchange platform and community of practice to foster collaboration among public health, data science, and technology experts worldwide. Since its inception in early 2022, the Innovation Forum has played a pivotal role in reshaping how epidemic intelligence practitioners and scientists access cross-disciplinary globally distributed innovations [2].

One of the primary challenges in pandemic intelligence is the fragmented nature of current efforts [1, 6, 7]. With a multitude of actors—governments, NGOs, philanthropic organizations, and private entities—investing in epidemic intelligence, siloed initiatives have hampered the effectiveness of global responses. The Innovation Forum directly addresses this by offering a collaborative space where stakeholders share insights, co-create solutions, and identify shared priorities. Through these regular interactions, the Forum cultivates a trust-based environment that encourages openness and enables collaborative innovation that transcends institutional boundaries.

The Innovation Forum’s unique approach emphasizes a holistic view of epidemic intelligence by covering a wide range of topics. From data aggregation and linkage, epidemic intelligence workforce development, and digital twins to standards and interoperability, climate and health, and citizen-generated data, each session encourages discussions that are critical to building a resilient epidemic intelligence framework. These sessions reflect a commitment to addressing complex, cross-cutting challenges that are often overlooked in traditional disease surveillance. This diversity in topics fosters innovation and invites global perspectives on pressing issues such as integrating citizen data into health policymaking, the implications of artificial intelligence for public health, and the role of climate data in anticipating health risks.

One of the Innovation Forum’s key strengths lies in the diversity and growing reach of its participants. What began as a small circle of professionals with existing collaborations with the WHO Hub staff has since expanded into a truly global network of specialists. Participants and speakers now come from a wide array of backgrounds and, as of December 2024, represent 43 countries across different regions. This diverse geographical representation is essential for ensuring that the Forum captures a comprehensive range of experiences and perspectives, enabling more inclusive and culturally responsive discussions. Furthermore, by tracking participant metrics, including gender diversity and representation from the Global South, the Forum actively promotes equitable engagement and strives to broaden the reach of epidemic intelligence to traditionally underrepresented regions.

Communication is also central to the Forum’s mission. Meeting reports published in this edition of BMC Proceedings serve as a valuable resource for the global public health community, disseminating insights and fostering a shared understanding of emerging health threats. The Forum’s benchmarks for inclusivity and diversity are not just symbolic; they are foundational to the success of epidemic intelligence, bringing in perspectives that enrich our collective understanding of global health challenges and solutions.

The WHO Pandemic Hub, through the Innovation Forum, has laid the foundation for a more coordinated, inclusive, and forward-looking global health intelligence network. As we look to the future, the Innovation Forum is developing collaborations with other networks, such as the Global Partnership for Sustainable Development Data, to create an even more extensive and richer opportunity for innovation sharing [8]. By supporting and expanding these collaborative efforts, we can pave the way for a resilient global ecosystem that not only anticipates but effectively fosters a human network of multi-disciplinary specialists who can work together to respond to the infectious disease challenges of tomorrow.

## Data Availability

Not applicable.

## Abbreviations

AI: Artificial Intelligence
COVID-19: Coronavirus Disease 2019
NGO: Non-Governmental Organization
SDG: Sustainable Development Goal
WHO: World Health Organization
